# Novel Polyoxyethylene-Containing Glycolipids Are Synthesized in *Corynebacterium matruchotii* and *Mycobacterium smegmatis* Cultured in the Presence of Tween 80

**DOI:** 10.1155/2011/676535

**Published:** 2010-07-20

**Authors:** Cindy Wang, Engy A. Mahrous, Richard E. Lee, Martha M. Vestling, Kuni Takayama

**Affiliations:** ^1^Mycobacteriology Research Laboratory, William S. Middleton Memorial Veterans Hospital, Madison, WI 53705, USA; ^2^Chemical Biology & Therapeutics, St. Jude Children's Research Hospital, Memphis, TN 38105, USA; ^3^Department of Chemistry, University of Wisconsin, Madison, WI 53706, USA; ^4^Department of Bacteriology, University of Wisconsin, Madison, WI 53706, USA

## Abstract

The addition of polyoxyethylene sorbitan monooleate (Tween 80) to a culture of mycobacteria greatly influences cell permeability and sensitivity to antibiotics but very little is known regarding the underlying mechanism. Here we show that *Corynebacterium matruchotii* (surrogate of mycobacteria) converts Tween 80 to a structural series of polyoxyethylenic acids which are then used to form novel series-2A and series-2B glycolipids. Minor series-3 glycolipids were also synthesized. The polyoxyethylenic acids replaced corynomycolic acids in the cell wall. Correspondingly the trehalose dicorynomycolate content was reduced. MALDI mass spectrometry, MS-MS, ^1^H-NMR, and ^13^C-NMR were used to characterize the series-2 glycolipids. Series-2A glycolipid is trehalose 6-C_36:2_-corynomycolate-6′-polyoxyethylenate and series-2B glycolipid is trehalose 6-C_36:2_-corynomycolate-6′-furan ring-containing polyoxyethylenate. *Mycobacterium smegmatis* grown in the presence of Tween 80 also synthesizes series-2 type glycolipids. The synthesis of these novel glycolipids in corynebacteria and mycobacteria should result in gross changes in the cell wall permeability and drug sensitivity.

## 1. Introduction

The cell wall of *Mycobacterium tuberculosis* is important to its virulence and intrinsic antimicrobial resistance. Several of the firstline antimycobacterial drugs target the enzymes involved in cell wall synthesis. Mycolic acids, long-chain *α*-alkyl, and *β*-hydroxy fatty acids are major characteristic constituents of a distinct group of Gram-positive bacteria of the *Corynebacterineae* which includes mycobacteria, corynebacteria, norcardia, and rhodococci. The mycobacterial and corynebacterial cell wall core is composed of mycolyl arabinogalactan-peptidoglycan complex which is surrounded by free lipids [1–3]. In addition, mycolic acids are also esterified to trehalose, glucose, and glycerol. Both cell wall-bound mycolates and mycolate-containing glycolipids are crucial part of the outer permeability barrier that confers low permeability and the intrinsic resistance to many antibiotics [1, 2]. 

The composition and amount of mycolic acids have been shown to affect the virulence, growth rate, colony morphology and permeability of *M. tuberculosis* [1, 2, 4, 5]. In *Corynebacterineae* trehalose dimycolate is the major structural constituents of cell wall glycolipids and trehalose monomycolate (TMM) is the carrier of mycolic acids during biosynthesis of the cell wall. *Corynebacterium glutamicum* defective in trehalose biosynthesis is unable to synthesize corynomycolate-containing glycolipids of trehalose monocorynomycolate (TMCM), trehalose dicorynomycolate (TDCM), and cell wall arabinogalactan-corynomycolates when sucrose is used as carbon source [6]. In a recent study, the content of cell wall corynomycolic acid was manipulated in a *C. glutamicum *mutant strain deficient in intrinsic trehalose synthesis by using different carbon sources [7]. Eradication or impaired cell wall corynomycolic acid synthesis resulted in increased susceptibility to antibiotics and augmented permeability to glycerol, indicating a crucial role of cell wall corynomycolic/mycolic acid content in maintaining the permeability barrier of *Corynebacterineae* [7].

Tween 80 is a nonionic, surface-active detergent often added to liquid media to reduce cell clumping and obtain homogeneous cell suspensions of mycobacteria. It has been shown that Tween 80 increases the sensitivity of *Mycobacterium avium* complex to antituberculosis drugs [8, 9]. The level of glycopeptidolipids was reduced with increase in Tween 80 cell concentration in the culture medium of the *M. avium-Mycobacterium intracellulare *complex [8]. Furthermore, Tween 80 caused elongation and inhibited the formation of fibrillar network material on the L1 layer [10]. It has been proposed that Tween 80 acts directly on the mycobacterial cell wall and subsequently alters its permeability [8]. These findings are consistent with the idea that Tween 80 increases cell-envelope permeability thereby enhancing drug penetrability. These results led to the recommendation that Tween 80 should not be used in drug susceptibility test media and results of cells that have been cultured in the medium containing Tween 80 should be interpreted with caution [9, 11]. Supplementation of culture medium with Tween 80 has been shown to trigger an increase in L-glutamate production by at least 10-fold in *C. glutamicum* ATCC 13869 [12]. The molecular basis of Tween-induced antimycobacterial susceptibility and L-glutamate overproduction remains unclear. 

In this study, we investigated the possibility that Tween 80 is used to synthesize novel glycolipids which could alter the cell wall permeability in *Corynebacterium matruchotii*, a model organism for mycobacteria. We compared the lipid profile of *C. matruchotii* grown in the presence and absence of Tween 80 and discovered that this nonionic detergent induced the synthesis of significant amounts of novel and structurally related glycolipids containing polyoxyethylene units (polyethylene glycol) derived from Tween 80. These glycolipids called series-2A and -2B are synthesized by the esterification of polyoxyethylenic acid with TMCM to form TDCM-like glycolipids. We also show that *Mycobacterium smegmatis* is able to synthesize polyoxyethylenate-containing glycolipid. The identification of these novel glycolipids has led us to suggest the mechanism of change in permeability and drug sensitivity of corynebacteria/mycobacteria caused by the presence of Tween 80 in the culture medium.

## 2. Materials and Methods

### 2.1. Bacterial Strains and Growth Conditions

*C. matruchotii *ATCC14266 and* C. glutamicum* ATCC13032 were obtained from American Type Culture Collection (Manassas, VA, USA). * C. matruchotii *was grown to late-log phase in brain heart infusion supplemented with 2% yeast extract in the presence or absence of 0.05% Tween 80. * C. glutamicum *was grown to late-log phase in Luria-Bertani medium in the presence or absence of 0.05% Tween 20.* M. smegmatis* mc^2^155 was grown to late-log phase in the glycerol-alanine-salts medium [13] plus and minus Tween 80. Corynebacteria were grown in the incubator/shaker at 30°C and 150 rpm while *M. smegmatis* was grown at 37°C and 150 rpm.

### 2.2. Preparation of Total Cellular Mycolic/Corynomycolic Acid Methyl Esters (MAME) and Fatty Acid Methyl Esters (FAME)

*C. matruchotii* and *M. smegmatis* (each at 200 mg wet weight) were saponified in 1.0 ml of 2 M KOH at 65°C for 3 hours with intermittent mixing. The mixtures were then acidified with HCl, extracted with 2.5 ml of chloroform/methanol (2 : 1), methylated with diazomethane and analyzed by silica gel thin layer chromatography (TLC).

### 2.3. Extraction of Total Lipids from Cells of Corynebacterium and Mycobacterium

*C. matruchotii*, *C. glutamicum,* and *M. smegmatis *(each at 100 g wet weight) were extracted with 500 ml of chloroform/methanol (2 : 1) at room temperature for 16 hours with stirring and the extracted residues were re-extracted twice. The pooled extracts were analyzed by TLC and used as the source of TMCM, TDCM, series-2 glycolipid, and series-3 glycolipid.

### 2.4. Preparation of the Cell Wall Skeleton

The delipidated cells of *C. matruchotii* and *C. glutamicum *(1.6 g dry wt.) were boiled in 15 ml of 1% SDS (sodium dodecyl sulfate), washed in ethanol and dried to yield the cell wall skeleton containing the peptidoglycan-arabinogalactan-corynomycolate complex.

### 2.5. Isolation and Purification of TMCM, TDCM, Series-2 Glycolipid, and Series-3 Glycolipid

 The chloroform/methanol extracts (1.5 g each) of *C. matruchotii *and *C. glutamicum* grown in the presence of Tween 80 or Tween 20 (for the latter) were dissolved in diethyl ether at 25 mg/ml, cooled to 4°C, two volumes of cold methanol were added and mixed. The supernatant containing the series-2 glycolipid, series-3 glycolipid and TMCM was separated from the precipitate containing the TDCM and a trace amount of TMCM by centrifugation. Each of the two fractions was then fractionated on a silica gel column (grade 62, Merck, 3.2 × 23 cm) using a stepwise gradient of one bed volume each of chloroform/methanol/concentrated ammonium hydroxide (CMN) (a) 90 : 10 : 1.5, (b) 85 : 15 : 1.5, (c) 80 : 20 : 1.5, (d) 70 : 30 : 1.5, and (e) 55 : 45 : 1.5 (v/v). About 6 ml fractions were collected and analyzed by TLC. From the supernatant, series-2 glycolipid was partially separated from series-3 glycolipid by using CMN (85 : 15 : 1.5). Elution of series-2 glycolipid was followed by that of series-3 glycolipid. Series-3 glycolipid is a minor component that is difficult to purify. From the precipitate, TMCM eluted from the silica gel column at CMN (80 : 20 : 1.5) whereas TDCM eluted at CMN (85 : 15 : 1.5). Each of these fractions was further fractionated on a Sephadex LH20 gel filtration column (2.1 × 150 cm) in chloroform/methanol (4 : 1) and analyzed by TLC and MALDI mass spectrometry. Silica gel column chromatography of the chloroform/methanol extracts without solvent precipitation yielded a mixture of series-2, series-3 glycolipids and TDCM in the CMN (85 : 15 : 1.5) effluent.

For the preparation of series-2-type glycolipid from *M. smegmatis*, the chloroform/methanol extract was precipitated in cold diethyl ether/methanol and carried through the two column fractionations (see above). Final purification was accomplished on a Sephadex LH20/LH60 mixed bed gel filtration column (2.1 × 150 cm) in chloroform/methanol (4 : 1).

### 2.6. Isolation and Purification of Corynomycolic Acids from the Cell Wall Skeleton

 The cell wall skeleton of *C. matruchotii* and *C. glutamicum* (0.8 g) was saponified in 2 M KOH and the free fatty acids were recovered as previously described. The fatty acids were methylated with diazomethane and the MAME was separated from FAME by silica gel column chromatography (1 × 22 cm) using a stepwise gradient of diethyl ether in hexane (25 ml). The purified MAME was analyzed by MALDI mass spectrometry.

### 2.7. Mild Base Hydrolysis of Series-2 Glycolipid and Cell Wall Skeleton

Purified polyoxyethylene-containing series-2 glycolipid (2 mg) was mixed with aqueous 15% tetrabutyl ammonium hydroxide (TBAH) (1 ml) and incubated at 100°C for 60 minutes [14]. To this mixture 1 ml of dichloromethane and 0.1 ml of iodomethane were added and incubated at room temperature with vigorous mixing for 30 minutes. The methyl esters in the reaction mixture were analyzed by MALDI mass spectrometry for the presence of methyl polyoxyethylenates.

 The cell wall skeletons (0.8 g dry wt) from *C. matruchotii* grown in the presence and absence of Tween 80 were treated with TBAH as described above. Then 0.3 ml of 1-iododecane was added, incubated further and the *n*-decanoyl esters were extracted with chloroform/methanol (2 : 1) and fractionated on a silica gel column using a stepwise gradient in diethyl ether in hexane. Pooled fractions were then analyzed by MALDI mass spectrometry for the presence of *n*-decanoyl polyoxyethylenates.

### 2.8. Analysis of Corynomycolic Acid and Sugar Carrier Derived from Purified Series-2 Glycolipid

Purified series-2 glycolipid (5 mg) was saponified in 1.0 ml of 2 M KOH as previously described [15]. This reaction mixture was acidified, 2.5 ml of chloroform/methanol (2 : 1) was added and mixed to yield a two-phase system. The lower organic layer contained the corynomycolic acid which was methylated with diazomethane, purified on a silica gel column and analyzed by TLC and MALDI mass spectrometry.

The upper aqueous layer was assayed for the presence of reducing sugar [16]. The presence of glucose was assayed before and after hydrolysis of the aqueous layer in 2 M trifluoracetic acid at 100°C for 2 hours using the glucose oxidase assay kit (Sigma, St. Louis, MO). The sugar in the aqueous layer was derivatized to the TMS-sugar with TMSI (N-trimethylsilyl imidazole, Pierce, Rockford, IL) and analyzed by TLC and MALDI mass spectrometry.

### 2.9. Silica Gel TLC

Silica gel GHL (0.25 mm, Analtech, Newark, DE, USA) was used for all TLC analyses. For the analysis of MAME and FAME the solvent system was petroleum ether/diethyl ether (7 : 1), for the analysis of TMCM, TDCM, and series-2 glycolipid the solvent system was chloroform/methanol/concentrated ammonium hydroxide (35 : 15 : 1.5) and for the analysis of TMS-derivatives of the sugar the solvent system was chloroform/methanol (98 : 2). The lipids were detected by spraying the TLC plate with 0.6% dichromate in 55% sulfuric acid followed by charring. The glycolipids were revealed by spraying the plate with Bial's reagent (Sigma-Aldrich, St. Louis, MO, USA) followed by heating at 110°C for 10 minutes.

### 2.10. Nuclear Magnetic Resonance Spectroscopy (NMR)

 Nine mg of purified series-2 glycolipid were dissolved in 600 *μ*l of CDCl_3_ (deuterated chloroform, Cambridge Isotope Laboratories, Cambridge, MA). One dimensional ^1^H-NMR and two dimensional ^1^H-^1^H-COSY, ^1^H-^1^H-TOCSY and ^1^H-^13^C-HSQC spectra of the sample were acquired using a 500 MHz Varian-INOVA NMR spectrometer equipped with a 5 mm triple resonance *trpfg* probe (Varian Inc., Palo Alto, CA). Signals in the NMR spectra were referenced to a tetramethylsilane (TMS) internal standard.

### 2.11. MALDI Mass Spectrometry

 MALDI experiments were performed on a Bruker Ultraflex III mass spectrometer (Billerica, MA) equipped with a SMART BEAM laser, a LIFT cell and Compass v. 1.2 software. The samples dissolved in chloroform/methanol (2 : 1 or 4 : 1) were placed on top of a thin layer of 2.5-dihydroxybenzoic acid on the stainless steel target. Calibration spots were prepared by placing PEG 1500 or PPG 1000 in methanol on top of a thin layer of 2,5-dihydroxybenzoic acid close to the sample location. Fragmentation (MS-MS) was generated by raising the laser power and the potential of the LIFT cell.

## 3. Results

### 3.1. Novel Glycolipids Are Present in the TDCM-Like Fraction of the Chloroform/Methanol Extract of *C. matruchotii* Grown in the Presence of Tween 80

**Figure 1:**
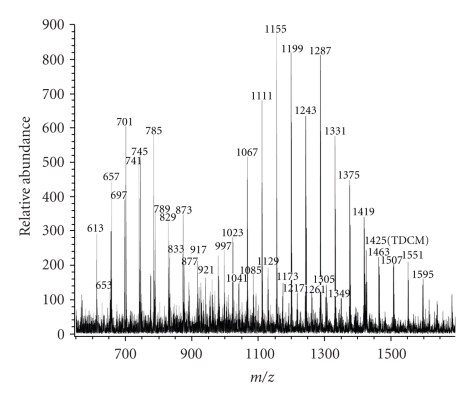
*Partial MALDI mass spectrum of TDCM-like glycolipid from C. matruchotii grown in the presence of Tween 80.* The glycolipids in the chloroform/methanol extract were purified by silica gel and Sephadex LH20 column chromatography. Molecular ion peaks (MNa^+^) at *m/z* 613 to 921 represent series-3 glycolipids and those at *m/z* 997 to 1595 represent series-2 glycolipids. TDCM (*m/z* 1425) represents a glycolipid containing two C_36:2_-corynomycolates.

 When we fractionated the chloroform/methanol extract of *C. matruchotii* grown in the presence of Tween 80 by a procedure that should yield TDCM (silica gel and Sephadex LH20 gel filtration column chromatography), the MALDI spectrum showed very little TDCM but instead revealed several series of novel TDCM-like glycolipids (Figure 1). We have named these glycolipids series-2A, -2B, -3A, and -3B. The MALDI spectrum shows overlapping series-2A (*m/z* 997 to 1349) and -2B (*m/z* 1023 to 1595) and overlapping series-3A (*m/z* 613 to 921) and -3B (*m/z* 653 to 917) molecular ion peaks. These peaks appeared at intervals of 44 atomic mass units (amu). The TDCM-like fraction from *C. matruchotii* grown in the absence of Tween 80 did not show these molecular ions.

### 3.2. A Comparison of the Corynomycolate Content in the Cell Wall Skeleton of *C. matruchotii* Grown in the Presence and Absence of Tween 80

 The synthesis of these novel glycolipids might affect the cellular corynomycolate content. The corynomycolate content in the cell wall skeleton of *C. matruchotii* grown in the presence and absence of Tween 80 was determined as MAME by MALDI mass spectrometry (Table 1). In the absence of Tween 80 in the culture medium, the MALDI spectrum showed the presence of a wide range of molecular ions for MAME from C_26:0_ to C_36:1_ with the most abundant molecular ions appearing at *m/z* 533 and 559 representing methyl C_32:0_-, and C_34:1_-corynomycolates, respectively. The cell wall skeleton of *C. matruchotii* grown in the presence of Tween 80 showed a single prominent methyl C_36:2_-corynomycolate presumably derived from the Claisen-type condensation of two molecules of oleic acid from Tween 80 [17]. Other molecular ions for methyl C_30:1_-, C_33:1_-, C_34:1_- and C_36:1_-corynomycolates were intermediate in intensity. The relative abundance of methyl C_32:0_-corynomycolate was low (<10).

### 3.3. Effect of Growing *C. matruchotii* and *C. glutamicum* in the Presence of Tween 80 and Tween 20 on the Corynomycolate Composition of Purified TMCM and TDCM

 The corynomycolate content of purified TMCM and TDCM from *C. matruchotii* grown in the presence of Tween 80 and *C. glutamicum* grown in the presence of Tween 20 was determined by MALDI mass spectrometry of the intact lipids as well as after saponification and methylation of the corynomycolic acids. The results showed that the major corynomycolate is C_36:2_ in both TMCM and TDCM of *C. matruchotii* and C_28:0_ in both TMCM and TDCM of *C. glutamicum*. This is similar to the corynomycolate composition of the cell wall skeleton of *C. matruchotii* as reported above. As in the cell wall skeleton, the C_36:2_-corynomycolate was not the major component in TMCM or TDCM of *C. matruchotii* grown in the absence of Tween 80. Thus the oleic acid derived from Tween 80 and myristic acid derived from Tween 20 are the probable sources of the C_36:2_- and C_28:0_-corynomycolates, respectively [18].

### 3.4. Purification and MALDI Analysis of Series-2 Glycolipid from *C. matruchotii*

 We have purified series-2 glycolipid from the chloroform/methanol extract of *C. matruchotii* grown in the presence of Tween 80. MALDI spectrum of this sample revealed the clear presence to two structural series of glycolipids (Table 2). The size range of the molecular ions of series-2A glycolipid was from *m/z* 953 to 1129 with the most abundant peak appearing at *m/z* 953. The molecular ions of series-2B glycolipid were from *m/z* 1023 to 1551 with the most abundant peak appearing at *m/z* 1155. The minor series-2A lipid differed from the major series-2B lipid by 26/18 amu. In each set the molecular ion peaks appeared at 44 amu intervals suggesting that the difference is the oxyethylene unit (O–CH_2_–CH_2_) derived from Tween 80.

### 3.5. Relative Distribution of TMCM, TDCM, Series-2 Glycolipid, and Cell Wall Skeleton Corynomycolic Acid in *C. matruchotii* Grown in the Presence and Absence of Tween 80

 The cellular content of TMCM, TDCM, series-2 glycolipid, and cell wall skeleton corynomycolic acid were quantified for *C. matruchotii* grown in the presence and absence of Tween 80. Cells grown in the presence of Tween 80 contained 0.18% TMCM, 0.08% TDCM, 0.41% series-2 glycolipid, and 0.08% cell wall skeleton corynomycolate (dry weight of cells). The series-3 glycolipid was not quantified due to their relatively low abundance and difficulty of purification. Cells grown in the absence of Tween 80 contained 0.18% TMCM, 0.40% TDCM, 0.0% series-2 glycolipid, and 0.93% cell wall skeleton corynomycolate. Thus in the presence of Tween 80, the synthesis of TDCM and incorporation of corynomycolic acid into the cell wall were depressed with the appearance of the novel series-2 and series-3 glycolipids. Similar differences were found in the TDCM and cell wall skeleton corynomycolate contents of *C. glutamicum* grown in the presence and absence of Tween 20.

### 3.6. Chemical Composition of Series-2 Glycolipid

 The purified series-2 glycolipid was saponified with 2 N KOH, acidified, the organic layer of chloroform/methanol/water two-phase system was methylated and analyzed by MALDI mass spectrometry. The spectrum showed a single prominent molecular ion peak (MNa^+^) at *m/z* 585 which represented methyl C_36:2_-corynomycolate (MAME). There was no evidence for the presence of FAME. Thus the fatty acid present in series-2 glycolipid is C_36:2_-corynomycolic acid.

Purified sample of series-2 glycolipid was saponified and the water soluble fraction was examined to determine the nature of the carrier of the lipids (corynomycolate and polyoxyethylenate) which was provisionally assumed to be a sugar. It was negative in the test for reducing sugar and after base hydrolysis, it was positive for glucose in the enzymatic glucose oxidase assay. We converted the sugar derived from series-2 glycolipid to the TMS-derivative and analyzed the product by TLC and MALDI mass spectrometry. This product comigrated with authentic TMS-trehalose on TLC. MALDI spectrum of the TMS-derivative showed molecular ion peaks at *m/z* 726, 798, 870 and 942 (Table 3). These peaks represented molecular ions with varying degree of derivatization of trehalose with hexa-TMS trehalose being the most prominent. These results are consistent with the carrier sugar being trehalose. 

**Figure 2:**
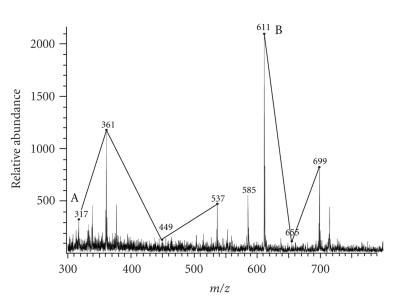
*MALDI mass spectrum of methyl polyoxyethylenates derived from purified series-2 glycolipid from C. matruchotii grown in the presence of Tween 80.* The free acids from TBAH-hydrolyzed series-2 glycolipid were esterified with methyl iodide and analyzed by MALDI mass spectrometry.

**Figure 3:**
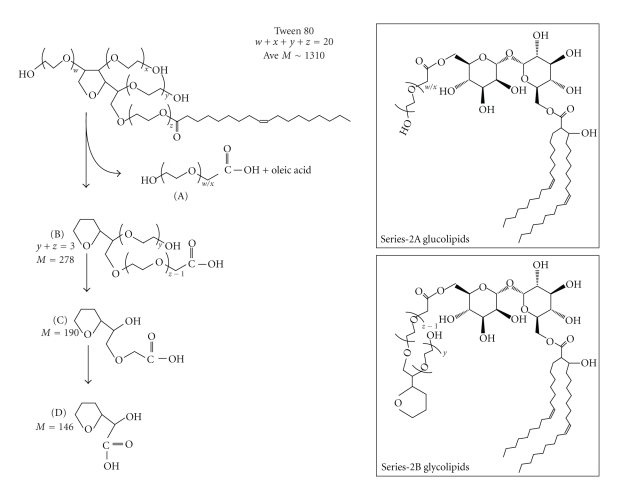
*Predicted products of degradation of Tween 80 by C. matruchotii. *Structure A is polyoxyethylenic acid derived from the furan ring of Tween 80 (http://www.sigma-aldrich.com/) and structure B is the furan ring-associated polyoxyethylenic acid. Structures C and D are further degradation products of B. The terminal hydroxyl group is thought to be oxidized to the carboxyl group and all of these products are used by the bacteria to synthesize series-2A and -2B glycolipids. Alternative to structure B, the carboxylic acid group could be on the other polyoxyethylenate unit. The proposed structures of series-2A and -2B glycolipids are shown in upper right and lower right panels, respectively.

We concluded that the series-2 glycolipid contains trehalose, C_36:2_-corynomycolic acid, and two structural series of polyoxyethylenic acids (which are the variables). We then attempted to isolate the polyoxyethylenic acids from series-2 glycolipid by mild base hydrolysis with TBAH. We were unsuccessful when we used the much stronger KOH reagent. Purified series-2 glycolipid was hydrolyzed in 15% TBAH at 100°C, the fatty acids were methylated and analyzed by MALDI mass spectrometry (Figure 2). The spectrum showed two series of molecular ion peaks occurring at intervals of 44 amu. They were MNa^+^ at *m/z* (A) 317, 361, 449, and 537; (B) 611, 655 and 699 as remnants of degradation. We could not associate the structures of these molecular ions with the polyoxyethylenic acids as shown in Figure 3. The molecular ion at *m/z* 585 represents methyl C_36:2_-corynomycolate. This is additional evidence that series-2 glycolipid contains polyoxyethylenate, replacing one corynomycolate in the structure of TDCM.

### 3.7. Proton and ^13^C-NMR Analysis of Series-2 Glycolipid

 The novel series-2 glycolipid is composed of trehalose, C_36:2_-corynomycolate, and polyoxyethylenate. We used 2D-NMR experiments to assign the proton and carbon chemical shifts for residues within the sample that corresponded to each of the three parts in the structure. The results are given in Tables 4 and 5. Signals from proton and carbon NMR were used to identify diagnostic biomarker peaks for trehalose, corynomycolate and polyoxyethylenate of series-2 glycolipid [19]. NMR analysis of the trehalose moiety gave the usual values for the chemical shift for H-1 through H-5 and C-1 through C-5 (Table 4). Of interest to us were the values of the chemical shifts of the methylene protons at position 6 where acylation might be expected to occur. The normal values for these methylene protons would be below 4.0 ppm. The observed downfield shift of these protons to 3.96 and 4.76 ppm is an indication that the C-6 position is acylated. This downfield shifting of resonances due to acylation of the sugar at the various positions is well established by observations on numerous sugar derivatives [20, 21]. These results are consistent with the structure of trehalose 6-C_36:2_-corynomycolate-6′-polyoxyethylenate where acylation occurs at both of C-6 and C-6′ positions. 

We have identified all of the protons and carbon resonances for all of the components of the corynomycolate and polyoxyethylenate in the series-2 glycolipid structure (Table 5). We identified the resonances of ^1^H and ^13^C associated with the two double bonds in the corynomycolate at 5.33 and 130.5 ppm, respectively. The ^1^H and ^13^C associated with the *α*-alkyl carbon gave resonances of 1.58 and 25.4 ppm, respectively. The ^1^H and ^13^C associated with the *β*-hydroxy carbon gave the expected resonances of 3.72 and 73.1 ppm, respectively. These NMR results further confirm the presence of corynomycolate in the series-2 glycolipid.

The proton and carbon resonances of key components of the proposed polyoxyethylenic acid (Figure 3) have been identified (Table 5). The ^1^H and ^13^C resonances associated with the fatty acyl *α*-position gave resonances of 2.34 and 34.3 ppm, respectively. The ^1^H and ^13^C signals associated with the oxyethylenic unit gave resonances of 3.64 and 70.7 ppm, respectively. The ^1^H and ^13^C signals associated with the furan ring structure of polyoxyethylenic acid have been identified within the sample. These results are consistent with the presence of polyoxyethylenic acyl group in series-2 glycolipid.

### 3.8. Biological Degradation of Tween 80 and the Generation of Polyoxyethylenic Acids

 We suggest that *C. matruchotii* metabolizes Tween 80 to form fragments containing carboxyl group that are used for the synthesis of series-2 glycolipid. We have deduced the probable structure of the polyoxyethylenate in series-2 glycolipid based on (a) the known structure of Tween 80, (b) molecular weights of series-2A and -2B glycolipids, (c) the requirement that the polyoxyethylenic acid contain a single hydroxyl group, and (d) our knowledge of the chemical composition of the series-2 glycolipid. The proposed structures of the polyoxyethylenic acid moiety of series-2 glycolipid are given in Figure 3. We suggest that two polyoxyethylene units are first removed from the furan ring of Tween 80 and oxidized to the polyoxyethylenic acid (structure A). The oleoyl acyl group is cleaved and converted to the C_36:2_-corynomycolic acid by the ortholog of Pks13 condensase from *M. tuberculosis* [17]. The remaining furan ring-containing polyoxyethylenic alcohol is converted to the acyl product to yield structure B. This product is further degraded in a stepwise manner to yield structures C and D. All of these polyoxyethylenic acids are then utilized by *C. matruchotii* for the synthesis of structural series-2A and-2B glycolipids.

### 3.9. MS-MS Analysis of Series-2B Glycolipid

**Figure 4:**
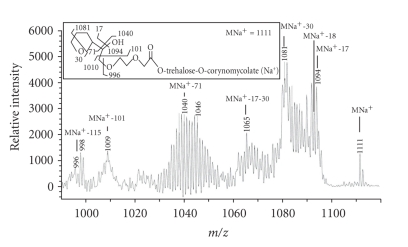
*MS-MS spectrum of the lifted MNa^+^ peak at m/z 1111.* Inset shows the structure and fragmentation pattern of this glycolipid from series-2B mass spectrum (Figure S1).

From the MALDI mass spectrum of purified series-2 glycolipids, the selected MNa^+^ peaks at *m/z* 1111 and 1331 (See Figure S1 in supplementary material online available at doi:10.1155/2011/676535) were lifted and submitted to MS-MS analysis. Figure 4 shows the MS-MS spectrum of the lifted MNa^+^ peak at *m/z* 1111, the proposed structure and the pattern of fragmentation. It shows molecular ion MNa^+^ peak at *m/z* 1111. It also showed MNa^+^-17 at *m/z* 1094, MNa^+^-18 at *m/z* 1093, MNa^+^-30 at *m/z* 1081, MNa^+^-17-30 at *m/z* 1064, MNa^+^-71 at *m/z* 1040, MNa^+^-101 at *m/z* 1010, and MNa^+^-115 at *m/z* 996. This is consistent with the given structure.

**Figure 5:**
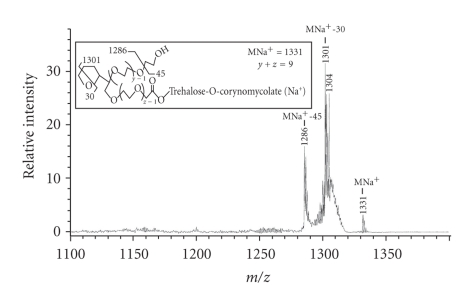
*MS-MS spectrum of the lifted MNa^+^ peak at m/z 1331.* Inset shows the structure and fragmentation pattern of this glycolipid from series-2B mass spectrum (Figure S1).

Figure 5 shows the MS-MS spectrum of the lifted MNa^+^ peak at 1331, the proposed structure and the pattern of fragmentation. It showed the molecular ion MNa^+^ peak at *m/z* 1331. It also showed MNa^+^-30 at *m/z* 1301 and MNa^+^-45 at *m/z* 1286. This is consistent with the given structure. The fragmentation of the *m/z* 1331 peak was not as extensive as that of the *m/z* 1111 peak.

### 3.10. Structures of Series-2A and -2B Glycolipids from *C. matruchotii*

 We suggest that series-2A glycolipid is trehalose 6-C_36:2_-corynomycolate-6′-polyoxyethylenate where the polyoxyethylenic acid is structure A as shown in Figure 3 (upper right panel). The series-2B glycolipid would be trehalose 6-C_36:2_-corynomycolate-6′-polyoxyethylenate where the polyoxyethylenic acids are structures B–D as shown in Figure 3 (lower right panel).

### 3.11. Preliminary Analysis of Series-3 Glycolipid

 We have established that series-3 glycolipid is composed of structural series-3A and -3B, is a minor component and is much smaller than the more prominent series-2 glycolipid. We also determined that it is composed of a sugar carrier and polyoxyethylenic acid but is devoid of corynomycolic acid. Although we were unable to arrive at a definite structure, the series-3 glycolipid might be the hypothetical trehalose-6-polyoxyethylenate which would be the precursor of series-2 glycolipid.

### 3.12. Evidence for the Presence of Polyoxyethylenate in the Cell Wall Skeleton of *C. matruchotii* Grown in the Presence of Tween 80

 When the cell wall skeleton of *C. matruchotii* grown in the presence of Tween 80 was saponified with 2 N KOH, methylated with diazomethane, and examined for the presence of methyl polyoxyethylenate by MALDI mass spectrometry, the spectrum was devoid of the characteristic molecular ion peaks appearing at 44 amu intervals. We considered the possibility that the labile polyoxyethylenic acid did not survive this harsh basic treatment or that this ester linkage is stable to base hydrolysis. Assuming that the former is the case, we tried a milder form of base hydrolysis. 

The cell wall skeleton of *C. matruchotii* grown in the presence and absence of Tween 80 were hydrolyzed in 15% TBAH and the free fatty acids were esterified with 1-iododecane to yield a more hydrophobic *n*-decanoyl rather than a methyl ester. These esters were purified on a silica gel column to obtain three pooled fractions: (a) nonpolar, (b) intermediate polarity, and (c) high polarity fractions. These six samples were analyzed by MALDI mass spectrometry.

MALDI spectrum of the nonpolar decanoyl esters from the cell wall skeleton of *C. matruchotii* grown in the presence of Tween 80 showed MNa^+^ molecular ion peaks for decanoyl corynomycolates. However, the molecular ion peaks at intervals of 44 amu which is characteristic of polyoxyethylenates were absent in all nonpolar and intermediate polarity decanoyl ester fractions.

**Figure 6:**
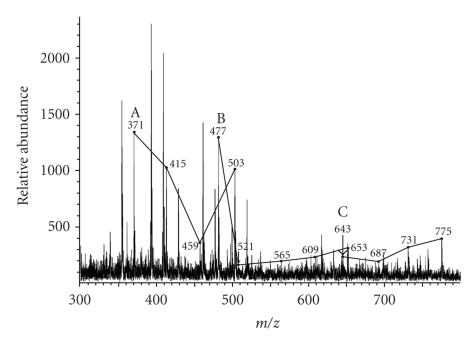
*MALDI mass spectrum of decanoyl polyoxyethylenates derived from the cell wall of C. matruchotii grown in the presence of Tween 80.* The free acids from TBAH-hydrolyzed cell wall skeleton were esterified with 1-iododecane, purified by silica gel column chromatography and analyzed by MALDI mass spectrometry. Three degraded series of decanoyl polyoxyethylenates are A, B, and C.

The MALDI spectrum of the high polarity decanoyl esters from the cell wall skeleton of *C. matruchotii* grown in the presence of Tween 80 showed three series of molecular ion peaks occurring at intervals of 44 amu. They were MNa^+^ at *m/z* (A) 371, 415, 459, and 503; (B) 477, 521, 565, 609, and 653; and (C) 643, 687, 731, and 775 (Figure 6). A similar sample from *C. matruchotii* grown in the absence of Tween 80 did not show such molecular ion series. Although the size ranges are consistent, we could not associate the structures of these molecular ions with the structure of polyoxyethylenic acids as shown in Figure 3. These could be degradation products with structural modification of the original polyoxyethylenates (to yield remnants). For example, the structural series A might be decanoyl polyoxyethylenate minus an ethyl group and series C might be decanoyl polyoxyethylenate minus an oxygen group. Based on these results we conclude that the cell wall skeleton of *C. matruchotii* grown in the presence of Tween 80 contains polyoxyethylenic acid, replacing the corynomycolic acid in the cell wall arabinogalactan. This is consistent with the observed low corynomycolate content in the cell wall skeleton of *C. matruchotii *grown in the presence of Tween 80.

### 3.13. *M. smegmatis* Can Synthesize Novel Series-2-Type Glycolipid

**Figure 7:**
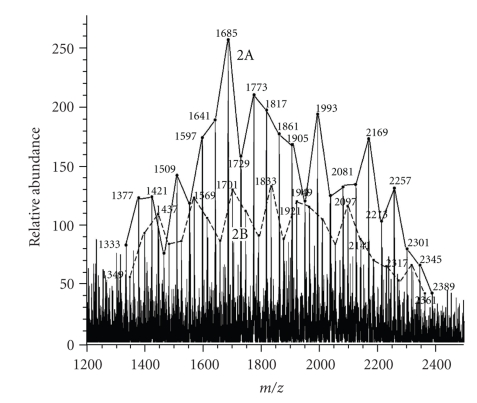
*MALDI mass spectrum of series-2-type glycolipid in the purified “TDM-like” lipid fraction of M. smegmatis grown in the presence of Tween 80.* 2A and 2B represent two structural series of polyoxyethylenate-containing glycolipids.

 We wanted to determine if *M. smegmatis* also metabolizes Tween 80 to the polyoxyethylenic acids and incorporate them into a sugar to form novel glycolipids. *M. smegmatis* grown in glycerol-alanine-salts/Tween 80 medium was extracted with chloroform/methanol and the extract was fractionated to yield the purified “trehalose dimycolate (TDM)-like” lipids. Such lipids are not found in *M smegmatis *grown in the absence of Tween 80. Its mobility on silica gel TLC was virtually identical to mycobacterial TDM. This sample was submitted to MALDI mass spectrometry and the spectrum is shown in Figure 7. It showed two structural series with the major series showing MNa^+^ peaks at *m/z* 1333 to 2389 with maximum abundance at *m/z* 1685. The minor series gave MNa^+^ peaks from *m/z* 1349 to 2361 with maximum abundance at *m/z* 1833. The peaks appeared at 44 amu intervals and the two structural series were off-set by 28 amu.

 We related these novel lipids from *M. smegmatis* to the series-2 glycolipid from *C. matruchotii*. The single variable in the structure must be the polyoxyethylenic acid and the two constants must be the sugar (assumed to be trehalose) and the fatty acid (as mycolic acid). The predominant fatty acid in this series-2-type glycolipid of *M. smegmatis* was found to be C_22:0_-mycolic acid. We suggest that the polyoxyethylenic acid (Figure 3, structure B) is elongated by the FAS-II system to increase the hydrocarbon chain length by 24 carbons in one case and 26 carbons in the other case [22]. The two modified structure B's are the variable components of series-2-type glycolipid with y+z ranging from 3 to 27. From this information the entire structural series can be tentatively defined as trehalose 6-C_22:0_-mycolate-6′-modified structure B. Although further work is needed to determine the precise structure, these results clearly demonstrates that *M. smegmatis *can also metabolize Tween 80 and use the degradation products for the synthesis of novel series-2-type glycolipids. Identical series-2-type glycolipids were found in the “TDM-like” fraction from *M. tuberculosis* H37Rv grown as submerged culture in Middlebrook 7H9 medium supplemented with 0.2% glycerol and 0.25% Tween 80 (K. Takayama, C. Wang and L. Armitige, unpublished results).

## 4. Discussion

These novel series-2A and series-2B glycolipids from *C. matruchotii *grown in the presence of Tween 80 were purified by solvent precipitation, silica gel column chromatography, and Sephadex LH20 column chromatography. They were then characterized by chemical analyses, MALDI mass spectrometry, MS-MS, ^1^H-NMR, and ^13^C-NMR. These glycolipids exist in structural series differing by 44 amu.

This study showed that *C. matruchotii *and* M. smegmatis* can absorb Tween 80, degrade it and use the degradation products (polyoxyethylenic acids and oleic acid) as building blocks to synthesize these novel glycolipids that becomes part of the cell envelope. The polyoxyethylenic acids replaces corynomycolic/mycolic acids in the synthesis of these predominant glycolipids and cell wall arabinogalactan-corynomycolate/mycolate. This is accompanied by marked reduction in TDCM and cell wall corynomycolic acid contents in *C. matruchotii*. Bacteria grown in the presence of Tween 80 are known to show gross changes in the cell's physical and biological properties.

The ability of these microorganisms to process Tween 80 did not evolve but rather is an adaptive response using existing biochemical machinery. So how do these microorganisms process Tween 80? We suggest that in *C. matruchotii*, Tween 80 is first transported into a cellular compartment called the periplasmic space where it is degraded to the polyoxyethylenic acids (structures A to D, Figure 3) and oleic acid. Oleic acid is transported into the cytoplasm where it is converted to C_36:2_-corynomycolic acid by Claisen-type condensation [17, 22]. It is then transported out of the cells and becomes trehalose 6-C_36:2_ corynomycolate (TMCM). The polyoxyethylenic acids in the periplasm are probably transferred to trehalose to form analogs of TMCM, trehalose 6-polyoxyethylenates. This hypothetical compound would be the substrate for the synthesis of series-2 glycolipid. In the case of *M. smegmatis*, the polyoxyethylenic acid (structure B, Figure 3) might be transported into the cytoplasm and enter the FAS-II system for the fatty acyl elongation reactions [22]. This elongated product is then transported outside the cells like mycolic acids, forms analogs of TMM and are used to synthesize the series-2-type glycolipid. *M. tuberculosis* H37Rv also seems to have this capability. 

The four probable reactions involved in the biological degradation of Tween 80 are (a) cleavage of oleate ester linkage by an esterase, (b) cleavage of the ether linkages and the formation of primary alcohol, (c) conversion of primary alcohol to aldehyde, and finally (d) oxidation of the aldehyde to carboxylic acid. Examples of all of these reactions in other microorganisms are found in the literature and a few are given below. 

The overall Tween 80-hydrolyzing activity of *M. smegmatis *was found to be strongly positive whereas that of *M. tuberculosis *was variable [23]. Mycobacteria are known to utilize oleic acid from Tween 80 as a carbon source [24]. A heat-stable Tween-hydrolyzing esterase with narrow substrate specificity was isolated and purified from *M. smegmatis* [25]. Others found that many of the rapidly growing mycobacteria produce esterase with Tween 80-hydrolyzing activity [26]. A Tween-hydrolyzing esterase was located on the surface of cell envelope of *M. smegmatis* and it was actively secreted into the culture medium [27]. These enzymes would generate oleic acid from Tween 80.

In the Gram-positive *Pseudonocardia* sp. strain K1, the ether bond in polyethylene glycol was cleaved by a Fe-dependent superoxide dismutase exhibiting diglycolic acid dehydrogenase/diglycolic acid oxidase activity [28, 29]. We have searched the corynebacteria and mycobacteria genomes and identified an enzyme annotated as Mn-dependent superoxide dismutase/*sod A*, which could cleave the ether bonds in Tween 80 to generate precursors to structures A and B (Figure 3).

The oxidation of the primary hydroxyl group in polyethylene glycol (PEG) to an aldehyde was demonstrated in *Sphingopyxis terrae* by a novel FAD-containing alcohol dehydrogenase encoded by *pegA *[30] and subsequently to the carboxylic acid by a novel NADP-containing nicotinoprotein aldehyde dehydrogaenase encoded by *pegC* [31]. The acyl-CoA ligase (*pegE*) catalyzes the ligation of PEG-carboxylate (polyoxyethylenate) and CoA on the cytoplasmic membrane [32]. We have identified homologs of PEG dehydrogenase (*pegA*), PEG-aldehyde dehydrogenase (*pegC*), acyl-CoA ligase (*pegE*), and AraC-type transcription regulator (*pegR*) in corynebacteria and mycobacteria genomes by sequence homology searches (*in silico *analysis).

There are examples of microorganisms utilizing various substrates to synthesize novel glycolipids with somewhat similar structure as series-2 and -3 glycolipids. *Rhodococcus *sp. strain MS11 grew well on a great number of *n*-alkanes [33]. When cultured on medium chain length *n*-alkanes (C_10_ to C_17_), strain MS11 produced biosurfactants lowering the surface tension of the cultures. The two major components of glycolipids consisted of trehalose esterified at the C-2 or C-4 position with a succinic acid and at the C-2′ position with a decanoic acid. *Rhodococcus opcus *1CP was found to utilize C_10_ to C_16_ *n*-alkanes as sole carbon sources [34]. Presumably the *n*-alkanes were converted to mycolic acids and incorporated into TDM. 

A number of studies have shown that the structure of the mycolic alkyl chains is affected by the nature of the carbon source [35–37]. When glucose, alkanes, polycyclic aromatic hydrocarbons or Luria-Bertani medium was used as the sole carbon source, polycyclic aromatic hydrocarbons-degrading *Mycobacterium* spp. LB501T, LB307T and VM552 produced varying mycolic acid profiles. Bacteria growing on poorly water-soluble substrates exhibited more hydrophobic mycolic acids than cells grown on glucose. This study demonstrated the importance of the growth substrate for mycolic acid profiling [37]. When *Mycobacterium frederiksbergense* LB501T was cultured under various substrate regimens, higher proportions of anthracene in the carbon source mixture led to higher cell surface hydrophobicities and more-hydrophobic mycolic acids [36]. The alkane-degrading *Rhodococcus erythropolis* strain E1 exhibited a clear correlation between the carbon source and the mycolic acid profiles [35]. Furthermore, more hydrocarbon-like compounds were exposed at the cell wall surface and strain E1 exhibited a radical change in cell wall permeability during growth on alkanes [35].

How the microorganism utilizes polyoxyethylenic acids to synthesize series-2 and -3 glycolipids remains unknown. Many of the genes and their encoded proteins involved in the synthesis and processing of TDCM/TMCM, TDM/TMM and cell wall corynomycolate/mycolate have been identified in corynebacteria/mycobacteria. Their functions were demonstrated by genetic manipulation and biochemical analysis [30]. These bacteria must be using the existing machinery including corynomycolyltransferases to transport and process polyoxyethylenic acids into series-2 and -3 glycolipids. 

Tween 80 used to supplement liquid culture medium was found to affect the formation of the L1 layer, which has been considered to be one of the pathogenic factors of *M. avium* complex [10]. It has been shown that cell wall mycolic acid content plays a crucial role in maintaining the permeability barrier of *Corynebacterineae *[7]. These results suggest that Tween 80 is far from an inert nonionic detergent and it causes induced changes in the cell envelope glycolipids and cell wall mycolate content that may contribute to increased cell envelope permeability and drug sensitivity in *M. tuberculosis*. This could also explain why *C. glutamicum* ATCC 13869 grown in the presence of Tween 40 overproduces L-glutamate [12]. Further we believe these changes to the cell wall are likely to have effects on macrophage uptake and the immune response to *M. tuberculosis*.

## Supplementary Material

Highly purified series-2 glycolipid prepared from the chloroform/methanol extract of *C. matruchotii* cultured in the presence of Tween 80 was analyzed by MALDI mass spectrometry. From the resulting spectrum (Figure S1), MNa^+^ peaks at *m/z* 1111 and 1331 were lifted for tendem mass spectrometry (MS-MS).
Figure S1: *MALDI mass spectrum of series-2 glycolipid from* C. matruchotii *grown in the presence of Tween 80*. This spectrum shows the structrual series of the major series-2B peaks at *m/z* 1067-1859 and minor series-2A peaks at *m/z* 1259-1567.

## Figures and Tables

**Table 1:** Molecular ions in MALDI mass spectra of MAME from the cell wall skeleton of *C. matruchotii* grown in the presence and absence of Tween 80.

Molecular ion:			Relative abundance^a^	
MNa^+^ (*m/z*)	*M*	Corynomycolate	no/Tween 80	w/Tween 80

449	426	C_26:0_	14	—
475	452	C_28:1_	12	—
477	454	C_28:0_	11	—
503	480	C_30:1_	—	34
505	482	C_30:0_	35	—
519	496	C_31:0_	37	—
531	508	C_32:1_	32	—
**533**	**510**	**C_32:0_**	**100**	—
545	522	C_33:1_	—	15
557	534	C_34:2_	17	—
559	536	C_34:1_	99	24
561	538	C_34:0_	10	—
**585**	**562**	**C_36:2_**	37	**100**
587	564	C_36:1_	21	26

^a^The most abundant molecular ions are given in bold face lettering. Molecular ion peaks with relative abundance of <10 were omitted.

**Table 2:** Molecular ions in MALDI mass spectrum of two structural series of purified series-2 glycolipid isolated from *C. matruchotii* grown in the presence of Tween 80.

Series-2A glycolipids:		Relative	Series-2B glycolipids:		Relative
MNa^+^(*m/z*)	*M*	abundance^a^	MNa^+^(*m/z*)	*M*	abundance^a^

**953**	**930**	**100**	1023	1000	32
997	974	50	1067	1044	58
1041	1018	31	1111	1088	74
1085	1062	27	**1155**	**1132**	**100**
1129	1106	29	1199	1176	93
—	—	—	1243	1220	73
—	—	—	1287	1264	93
—	—	—	1331	1308	65
—	—	—	1375	1352	51
—	—	—	1419	1396	32
—	—	—	1463	1440	25
—	—	—	1507	1484	23
—	—	—	1551	1528	24

^a^The most abundant molecular ions are given in bold face lettering.

**Table 3:** Molecular ions from MALDI mass spectrum of TMS derivative of trehalose derived from purified series-2 glycolipid of *C. matruchotii* grown in the presence of Tween 80.

MNa^+^(*m/z*)	*M*	Tentative identification	Relative abundance
726	703	Trehalose(TMS)_5_	18
798	775	Trehalose(TMS)_6_	100
870	847	Trehalose(TMS)_7_	23
942	919	Trehalose(TMS)_8_	13

Series-2 glycolipid from *C. matruchotii* was hydrolyzed under basic condition and the aqueous layer in the chloroform/methanol/water two-phase system was desalted. The sugar was converted to their TMS derivative and analyzed by MALDI mass spectrometry.

**Table 4:** Chemical shifts and assignment of proton and ^13^C in NMR of the trehalose moiety of purified series-2 glycolipid isolated from *C. matruchotii* grown in the presence of Tween 80.

Proton	Chemical shift (ppm)	^13^C	Chemical shift (ppm)
H-1	5.05	C-1	94.3
H-2	3.64	C-2	71.9
H-3	3.88	C-3	92.9
H-4	3.31	C-4	71.7
H-5	4.21	C-5	70.0
H-6, H-6′	3.96, 4.76	C-6	64.6

**Table 5:** Chemical shifts and assignment of proton and ^13^C in NMR of corynomycolate and polyoxyethylenate moieties of purified series-2 glycolipid isolated from *C. matruchotii* grown in the presence of Tween 80.

	Chemical shift	
	Proton (ppm)	^13^C (ppm)

C_36:2_-Corynomycolate^a^		
Terminal –**CH_3_**	0.84	14.0
–**CH_2_**–	1.28	29.4
–**CH**=**CH**–	5.33	130.5
–C(O) –**CH**(CH_2_–)–	2.42	52.4
–C(O) –CH(**CH_2_**–)–	1.58	25.4
–**CH**(OH) –	3.72	73.1
–CH=CH–**CH_2_**–	1.96	27.4
–**CH_2_**–CH–OH–	1.38, 1.56	34.1
Polyoxyethylenates^b^		
–**CH_2_**–C(O)O–	2.32	34.3
–O**CH_2_**–CH_2_–O–	3.64	70.7
–**CH_2_**– (OCH_2_–CH_2_–O)_m−1^−^_	5.19	70.2
–**CH_2_**– (OCH_2_–CH_2_–O)_n−1^−^_	4.60, 4.38	62.9
–O**CH_2_**– (ring)	3.71	61.7
–O**CH**– (ring)	3.63	69.3
–OCH_2_–**CH_2_**–CH_2_– (ring)	1.93, 2.01	32.8
–OCH–CH_2_–**CH_2_**– (ring)	2.06	33.1

^a^Structure of C_36:2_-corynomycolate:

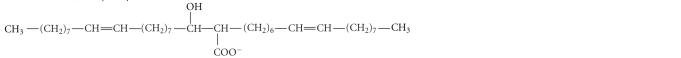

^b^The structures of polyoxyethylenates are shown in Figure 3.
