# Supplementary figures and images for: Novel Polyoxyethylene-Containing Glycolipids Are Synthesized in Corynebacterium matruchotii and Mycobacterium smegmatis Cultured in the Presence of Tween 80
Source: J Lipids. 2010 Jul 20;2011:676535. doi: 10.1155/2011/676535 (PMC3066834; doi:10.1155/2011/676535)

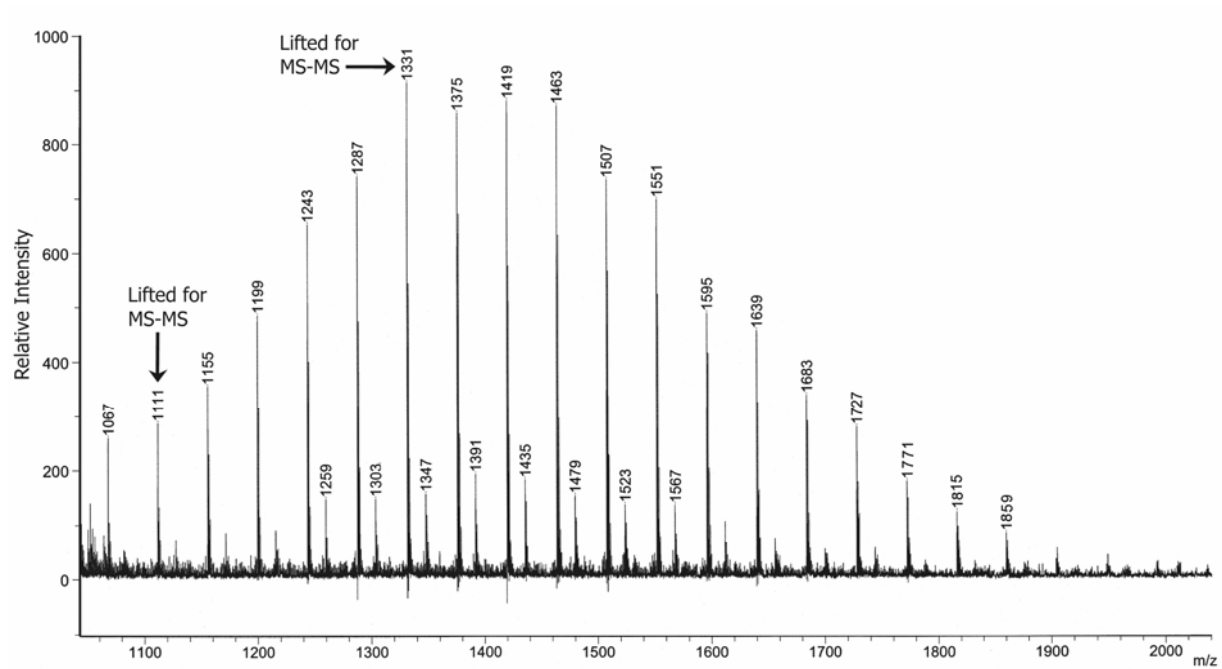

Figure S1

Supplement: Supplementary file 1 — Highly purified series-2 glycolipid prepared from the chloroform/methanol extract of C. matruchotii cultured in the presence of Tween 80 was analyzed by MALDI mass spectrometry. From the resulting spectrum (Figure S1), MNa+ peaks at m/z 1111 and 1331 were lifted for tendem mass spectrometry (MS-MS). Figure S1: MALDI mass spectrum of series-2 glycolipid from C. matruchotii grown in the presence of Tween 80. This spectrum shows the structrual series of the major series-2B peaks at m/z 1067-1859 and minor series-2A peaks at m/z 1259-1567. [file 676535.f1.pdf]
